# Validity of the Aboriginal Children’s Health and Well-being Measure: Aaniish Naa Gegii?

**DOI:** 10.1186/s12955-015-0351-0

**Published:** 2015-09-17

**Authors:** Nancy L. Young, Mary Jo Wabano, Koyo Usuba, Brenda Pangowish, Mélanie Trottier, Diane Jacko, Tricia A. Burke, Rita G. Corbiere

**Affiliations:** Laurentian University, Sudbury, ON Canada; Nahndahweh Tchigehgamig Wikwemikong Health Centre, Wikwemikong Unceded Indian Reserve, ON Canada; Nadmadwin Mental Health Clinic, Wikwemikong Unceded Indian Reserve, ON Canada; Elder, Wikwemikong Unceded Indian Reserve, ON Canada

**Keywords:** Aboriginal, Children, Well-being, Interviews, Questionnaire

## Abstract

**Background:**

Aboriginal children experience challenges to their health and well-being, yet also have unique strengths. It has been difficult to accurately assess their health outcomes due to the lack of culturally relevant measures. The Aboriginal Children’s Health and Well-Being Measure (ACHWM) was developed to address this gap. This paper describes the validity of the new measure.

**Methods:**

We recruited First Nations children from one First Nation reserve in Canada. Participants were asked to complete the ACHWM independently using a computer tablet. Participants also completed the PedsQL. The ACHWM total score and 4 Quadrant scores were expected to have a moderate correlation of between 0.4 and 0.6 with the parallel PedsQL total score, domains (scale scores), and summary scores.

**Results:**

Paired ACHWM and PedsQL scores were available for 48 participants. They had a mean age of 14.6 (range of 7 to 19) years and 60.4 % were girls. The Pearson’s correlation between the total ACHWM score and a total PedsQL aggregate score was 0.52 (*p* = 0.0001). The correlations with the Physical Health Summary Scores and the Psychosocial Health Summary Scores were slightly lower range (*r* = 0.35 *p* = 0.016; and *r* = 0.51 *p* = 0.0002 respectively) and approached the expected range. The ACHWM Quadrant scores were moderately correlated with the parallel PedsQL domains ranging from *r* = 0.45 to *r* = 0.64 (*p* ≤ 0.001). The Spiritual Quadrant of the ACHWM did not have a parallel domain in the PedsQL.

**Conclusions:**

These results establish the validity of the ACHWM. The children gave this measure an Ojibway name, *Aaniish Naa Gegii*, meaning “how are you?”. This measure is now ready for implementation, and will contribute to a better understanding of the health of Aboriginal children.

## Background

Population health research is a powerful approach to understanding the health needs of Canadians and this approach must include Aboriginal children [1]. Surveys such as the First Nations Regional Longitudinal Health Survey (RHS) [2], Aboriginal Children’s Survey (ACS) [3], Canadian Community Health Survey (CCHS) [4], and others are a starting point, and provide health estimates at a national level. Statistics from these and other sources document the significant health inequities that Aboriginal children experience in comparison to their mainstream peers [5, 6]. In 2009, First Nations communities in Canada ranked 68th on the Human Development Index while residing in a country that ranked 3rd internationally [5]. While these inequities have been recognized for many years, viable solutions remain elusive.

A critical challenge shared by Aboriginal health directors on- and off-reserve, is that they must manage health services at a local level, yet have limited data that is meaningful at this level. National surveys and analyses of health services utilization rates are extremely valuable to describe health at a national level, but lack the precision necessary to match services to needs at the local community level. Furthermore, the time between data collection and sharing of results is often several years, thus rendering these data ineffective for guiding program decisions. Ball [7] and Fremantle et al. [8] have argued that high quality data is an essential precursor to understanding the problems, identifying solutions, and improving health equity for Aboriginal children in Canada. This data must provide detailed health information that is meaningful at the local level, if we are to identify solutions [9–13].

The Aboriginal Children’s Health and Well-being Measure (ACHWM) was developed to meet the needs of local communities to assess their children’s health. We built upon our own foundational research in child health measurement [14, 15] and a strong partnership between Aboriginal health leaders and academic researchers in northeastern Ontario [16, 17].

The ACHWM was developed in collaboration with Aboriginal children, based on consultations with community partners and leading experts in the field, and informed by the literature [18]. The ACHWM is a generic health status profile according to the taxonomy proposed by Fryback in 2010 [19]. As such, its primary value is to generate a comprehensive summary of health that incorporates multiple domains and goes beyond the assessment of disease impacts [19]. The ACHWM’s development was grounded in an Anishinaabek conceptualization of “health” based on the Medicine Wheel [20, 21]. The Wheel’s quadrants represent spiritual, emotional, physical, and mental health. This model was also selected as the framework for the RHS [22]. While many Aboriginal communities in Canada share the general concepts within the Medicine Wheel [20–25], the nomenclature differs. The essence of this model and its relationship to health were easy for children to understand in our previous research [18].

The ACHWM is intended to be completed independently by children between the ages of 8 to 18 years [18]. There is significant evidence in the literature to support the validity of self-report by children [14, 26–28]. Child self-report is important to capture children’s perspectives of health, and is essential to promote feasibility and sustainability of the survey implementation process in a cost-efficient way. We have confirmed its content validity through detailed interviews with children and parents [29–31]. The construct validity has not yet been confirmed.

Five guiding principles were foremost in the development of the ACHWM. We sought to ensure that it: reflected health from an Indigenous world view; encompassed the full spectrum of health from illness-to-wellness and across the 4 quadrants of health identified in the Medicine Wheel; had the potential to empower children to share their own perspectives on health; was feasible for health leaders to implement and sustain; and contained sufficient detail to inform healthcare planning at the local level.

In summary, the ACHWM was created to augment collective experiential knowledge. It was intended to provide local health leaders with detailed information on children’s health status, guide the development of evidence informed healthcare planning at the local level, and support ongoing evaluation of program effectiveness. The innovative melding of a culturally grounded measure with a community driven survey offers the promise of putting high quality data in the hands of Aboriginal health leaders. A key requirement for the measure to achieve these objectives is for it to be valid.

The purpose of this paper is to report on the construct validity of the Aboriginal Children’s Health and Well-being Measure (ACHWM) in comparison to a mainstream measure of health-related quality of life and to a single global rating of health.

## Methods

A community-based collaborative research approach was utilized to assess the validity of the ACHWM in one First Nations community in northeastern Ontario. The process began by consulting with healthcare providers, community leaders, and Elders. The resulting proposal was approved by the First Nation’s Chief and Council, the Manitoulin Anishinaabek Research Review Committee, and the Laurentian University Research Ethics Board.

The term validity has been defined by many authors, often using slightly different operational definitions. We had previously established that the ACHWM had content validity based on the results of focus groups with Elders, experts, and children who confirmed that the questions represented the construct of health and well-being consistent with an Anishinaabek perspective. The Anishinaabek framework for “health” was based on the Medicine Wheel [20, 21]. The content validity was confirmed by detailed interviews with nine children and nine caregivers on the Wikwemikong Unceded Indian Reserve [31]. The current research sought to establish the measure’s concurrent construct validity in comparison to a known measure of a related concept: health-related quality of life from a mainstream perspective.

The population of interest was Aboriginal children living on-reserve. There is currently no gold-standard survey to assess the health and well-being in this population. The RHS [2] is the most applicable survey, but focuses on health behaviors rather than an individual’s perceptions of their health and well-being. The ACHWM was developed from the perspectives of children, who defined health and well-being through the development of questions that focused on feelings and perceptions rather than health behaviors (e.g., smoking, drinking, and drug use). This focus, on how the children are feeling, had the added benefit of enabling the questions to be framed both positively and negatively, which is important in the Anishinaabek view of balance. Thus, we looked to the literature for a mainstream measure that: focused on feelings and perceptions; could be completed by self-report; and was considered acceptable to the community.

### Measures

The primary outcome measure in this study was the ACHWM. We identified 3 potential child self-report measures to which to compare the ACHWM: the PedsQL [32, 33]; the KidScreen52 [34]; and a single Self-Rating of Health. We considered implementing all 4 measures (the ACHWM, PedsQL, KidScreen and Self-Rated Health), but elected to limit the number of questions to minimize the respondent burden. We elected to include the singe question on Self-Rated Health that has been commonly used in health research (*In general would you say your health is excellent*, *very good*, *good*, *fair*, *or poor*?) [35], plus one generic multi-item mainstream questionnaire: the PedsQL.

The PedsQL was selected as the preferred generic multi-item measure because it had been extensively tested, had excellent reliability and validity, and extensive normative data [36]. The PedsQL had also been previously implemented in school-based surveys [37]. The PedsQL had its origins in the United States, had been adapted for a variety of different languages and cultures, and was stable across cultures [38]. Furthermore, the PedsQL had an age range that matched the age range of the ACHWM: 8 to 18 years. These were key criteria for selection. Moreover, the PedsQL had been used in this First Nations community for an unrelated study in the past and was known to be acceptable to the community members. The resulting data would then provide benchmarks to aid in interpretation of the survey results and enable the assessment of the validity of the ACHWM.

### Sampling frame

We recruited First Nations children and youth from one First Nations reserve in Canada: the Wikwemikong Unceded Indian Reserve [39, 40]. The eligible age range was defined as 8 to 18 years to match the intended age group for the ACHWM [18]. Participants were recruited on-reserve at 3 special community events. Members of the community who lived on-reserve part-time were permitted to participate. Non-First Nation children and youth were not included in this study. Informed consent was obtained for all participants. In the community, the minimum age of child-consent was set at 12 years by Chief and Council. Parent consent and child assent were required for those under 12 years of age. All consent and assent forms included an agreement to meet with a mental health worker, if requested, following the survey.

### Data collection

Children were asked to complete the ACHWM independently using a computer tablet that enabled those with low literacy levels to participate via a text-to-speech option. At the end of the ACHWM researchers were able to access a brief screening report the tablet produced (note: access to the screening report was restricted by a password). Any participants who were identified as potentially needing clinical support were immediately seen by a member of the local mental health team to assess their needs. Participants also completed the 2 mainstream measures as benchmarks: (1) the PedsQL [32, 41]; and (2) a single Self-Rating of Health [35].

### Analysis

The ACHWM analysis began by computing an ACHWM Summary Score that included all items. In addition, we computed scores for each of the quadrants of the Medicine Wheel: Spiritual, Emotional, Physical and Mental Quadrant Scores. For the PedsQL, we computed a Total Score that included all items, 2 health Summary Scores (Psychosocial and Physical) and 4 Domain Scores^1^ (Physical, Emotional, Social, and School Functioning). The Self-Rating of Health was transformed to a binary variable where excellent and very good response options were coded as *better* health; and good, fair and poor response options were coded as *worse* health to address small cell frequencies and ensure confidentiality. The normality of the distribution of these scores were assessed using the Shapiro-Wilk test for normality and were considered to be non-normal if skew or kurtosis ratios exceeded ±3.0 in magnitude.

Three hypotheses were set a priori to test the validity of the ACHWM:

1. that the ACHWM Summary Score would correlate between 0.4 and 0.6 with the PedsQL Total Score, assessed using a Pearson’s correlation coefficient;
  Note that we interpreted the strengths of the Pearson’s correlations as recommended by Cohen, in which a correlation of 0.3 to 0.5 is considered moderate and above 0.5 is considered strong [42].
2. that the Physical, Emotional and Mental Quadrant Scores within the ACHWM would have stronger correlations with the related PedsQL Summary Scores and Domain Scores than with unrelated Summary Scores and Domain Scores, assessed using a Pearson’s correlation matrix;
  Note that we did not predict a significant correlation between the ACHWM Spiritual Quadrant score and any of the PedsQL summary or domains, because a key part of the justification for developing the ACHWM was the absence of spirituality in existing measures. However, the conceptual framework of the Medicine Wheel guides us to believe that all aspects of health are inter-related; we hypothesized that the Spiritual Quadrant should have a moderate correlation with the average of the ACHWM questions after excluding the Spiritual questions.
3. that those with *better* self-rated health (excellent or very good) would have significantly higher ACHWM Summary Scores than those with *worse* self-rated health (good, fair or poor),s assessed by an un-paired *t*-test.

## Results

All participants were recruited between August 20th 2013 and April 24th 2014 and completed the ACHWM, PedsQL, and SRH. These measures were completed at a variety of different locations in Wikwemikong: 18 were completed at a booth set up at the local arena during the summer break; 10 at a wellness promotional day; 9 at a mental health team event at the local health center; 7 at a booth at the local arena during a school professional activity day (student holiday); and 4 at a parent-engagement event at the local health center. In total, paired ACHWM and PedsQL data were available for 48 participants with a mean age of 14.6 (range of 7 to 19) years. Sixty percent of participants were girls. Note that 4 of the 48 participants were outside the intended target range but were not excluded: one was 7; and three were 19 years of age. The 7-year old was determined to be capable of self-report and the 3 older youth were all attending high school and therefore representative of the school-age population.

### Distribution of scores

All scores were normally distributed. The mean ACHWM Summary Score was: 71.4 (SD 11.5). The mean ACHWM Quadrant Scores were: Spiritual 75.7 (SD 16.2); Emotional 71.0 (SD 14.0); Physical 76.3 (SD 13.8); and Mental 58.5 (SD 12.2). These mean scores are shown in Table 1, by gender. Boys appeared to have higher ACHWM Total Scores and Quadrant Scores compared to girls. Age was not correlated with the ACHWM Summary Scores or Quadrant Scores (range of correlations between *r* = −0.10 and *r* = 0.06, *p* > 0.5), with the exception of the Spiritual Quadrant scores, which declined with age (*r* = −0.30, *p* = 0.038).

**Table 1:** Mean ACHWM scores by gender

ACHWM	Boys (*n* = 19)	Girls (*n* = 29)
Summary score	75.0 (SD 8.78)	69.0 (SD 12.60)
Spiritual quadrant scores	76.8 (SD 16.15)	74.9 (SD 16.50)
Emotional quadrant scores	75.4 (SD 10.89)	68.0 (SD 15.12)
Physical quadrant scores	83.0 (SD 9.45)	72.0 (SD 14.55)
Mental quadrant scores	60.5 (SD 11.22)	57.2 (SD 12.90)

The mean PedsQL Total Score was: 71.1 (SD 12.2). The mean PedsQL Summary Scores were: Psychosocial 66.8 (SD 14.4); and Physical Function 79.1 (SD 14.0). The distributions of Domains (or Scale Scores) were: Emotional Functioning 60.0 (21.1); Physical Functioning 79.1 (14.0); School Functioning 79.3 (15.3); and Social Functioning 61.0 (18.3). We noted that 19 % of participants had ceiling effects on one or more aspects of the PedsQL where ceiling effects extremely rare on the ACHWM (2 %).

### Hypothesis 1: that the ACHWM Summary Score would correlate moderately (0.4 to 0.6) with the PedsQL Total Score

**Fig. 1:**
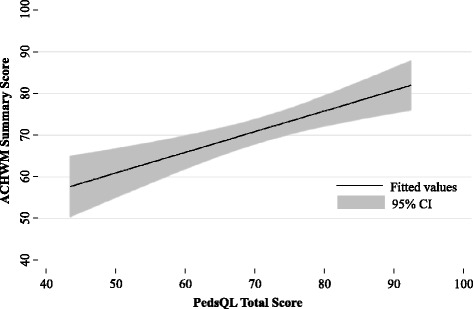
Relationship between the ACHWM summary score and PedsQL total score

The Pearson’s correlation between the ACHWM Summary Score and the PedsQL Total Score was 0.52 (95 % Confidence Interval 0.28–0.70, *p* = 0.0001). This relationship is depicted in Fig. 1. This finding supports the concurrent validity of the ACHWM Summary Score.

### Hypothesis 2: that ACHWM Quadrant Scores would have higher correlations with related PedsQL Domains than unrelated PedsQL Domains

We calculated Pearson’s correlation coefficients between the ACHWM Quadrant scores and the PedsQL summary and domain (scale) scores. These were guided by the a priori hypothesis of stronger correlations between similar constructs. The correlations are presented in Table 2. Note that the bold text in the table indicates cells where the highest correlations were expected a priori. The correlations between the total ACHWM scores with the PedsQL Summary Scores were stronger for the Psychosocial Health Summary Score (*r* = 0.51, 95 % CI 0.26–0.70, *p* = 0.0002) than the Physical Health Summary Score (*r* = 0.35, 95 % CI 0.07–0.57, *p* = 0.016).

**Table 2:** Correlation between ACHWM and PedsQL scores

			ACHWM				
			Quadrant scores				Summary score
			Spiritual^a^	Emotional	Physical	Mental	
PedsQL	Domain scores	Emotional functioning	*r* = 0.10	***r*** = **0.64**	*r* = 0.48	*r* = 0.60	*r* = 0.55
			*p* = 0.4979	***p*** < **0.0001**	*p* = 0.0006	*p* < 0.0001	*p* = 0.0001
		Physical functioning	*r* = 0.065	*r* = 0.39	***r*** = **0.45**	*r* = 0.21	*r* = 0.35
			*p* = 0.6613	*p* = 0.0063	***p*** = **0.0015**	*p* = 0.1549	*p* = 0.0159
		School functioning	*r* = 0.12	*r* = 0.48	*r* = 0.27	***r*** = **0.55**	*r* = 0.42
			*p* = 0.4125	*p* = 0.0005	*p* = 0.0683	***p*** = **0.0001**	*p* = 0.0029
		Social functioning	*r* = −0.08	*r* = 0.25	*r* = 0.034	*r* = 0.34	*r* = 0.1507
			*p* = 0.6119	*p* = 0.0911	*p* = 0.8195	*p* = 0.0174	*p* = 0.3065
	Summary score	Physical health summary	*r* = 0.07	*r* = 0.39	***r*** = **0.45**	*r* = 0.21	*r* = 0.35
			*p* = 0.6613	*p* = 0.0063	***p*** = **0.0015**	*p* = 0.1549	*p* = 0.0159
		Psychosocial health summary	*r* = 0.08	***r*** = **0.61**	*r* = 0.36	***r*** = **0.65**	*r* = 0.51
			*p* = 0.6107	***p*** < **0.0001**	*p* = 0.0113	***p*** < **0.0001**	*p* = 0.0002
	Total scale score		*r* = 0.08	*r* = 0.62	*r* = 0.46	*r* = 0.58	***r*** = **0.52**
			*p* = 0.5744	*p* < 0.0001	*p* = 0.0011	*p* < 0.0001	***p*** = **0.0001**

Shading and bold denotes a priori expectation for a higher correlation than with other cells

^a^Low correlations were expected with the Spiritual Quadrant, because this is not included in the PedsQL

Three of the Quadrant scores correlated significantly with their associated PedsQL domains, confirming the discriminant validity of three of the Quadrant scores. These were very close to the anticipated range of 0.4 to 0.6 in their strength of association. The Spiritual Quadrant was an exception, because there was no PedsQL domain related to this construct. Thus, the weak correlation confirms the unique contribution of this quadrant relative to the PedsQL and its discriminant validity. We explored the relationship between the Spiritual Quadrant and the aggregate score derived from the remaining questions, based on the Anishinaabek conceptualization that good health is requires balance across the quadrants. The moderate correlation of 0.51 (*p* = 0.0002) offers support for the validity of the Spiritual Quadrant.

### Hypothesis 3: Higher ACHWM scores for those with better Self-Rated Health (SRH)

We obtained SRH from 45 of our 48 participants. In this sample: 12 (26.7 %) rated their health as excellent; 9 (20.0 %) rated their health as very-good; 14 (31.1 %) rated their health as good, and 10 (22.2 %) rated their health as fair or poor. The *better health* group (pooled group of those with excellent and very good health) had a mean age of 14.9 (SD 2.61) years and mean ACHWM score of 75.2 (SD 11.77) and a mean PedsQL score of 76.9 (SD 10.68). The *worse health* group (pooled group of those with good, fair and poor health) had a mean age of 14.2 (SD 3.80), a mean ACHWM score of 67.2 (SD 10.41) and a mean PedsQL score of 64.2 (SD 10.08). The un-paired *t*-test value for the ACHWM between the *better health* group and *worse health* group was 2.43 (*p* = 0.019). For comparison, the un-paired *t*-test value for the two-group comparison with the PedsQL was 4.09 (*p* = 0.0002). These findings support the known-groups validity of the ACHWM.

An important part of this collaborative research project was ensuring a process responding to children whose answers on the survey required follow-up. Our response process was designed and fully supported by the local mental health team and proved a very important step in protecting the children who engaged in the survey. In our validation sample: 22 children required a brief assessment, many of whom required further follow-up; most of these children were already connected with services. This information is helpful to planning implementation in this and other communities.

## Discussion

This paper reports the validation of a new culturally grounded measure of health and well-being for Aboriginal children in Canada. The SRH values from this group are important to understand the natural distribution of health in our sample. In our sample: 26.7 % rated their health as excellent; 20.0 % rated their health as very-good; 31.1 % rated their health as good; and 22.2 % rated their health as fair or poor. These ratings are worse than those reported by the First Nation Information Governance Centre [43] who found 30.6 % to have excellent; 34.8 % very good; 27.1 % good; and only 7.5 % as fair/poor. The difference in scores may be due to the inclusion of parent-reported ratings in the national sample, whereas our sample was restricted to children’s self-report. Parent ratings in non-clinical samples have been shown to have higher scores than child-reported scores [44].

Validity was assessed through the testing of 3 hypotheses that were established a priori and outlined in the methods. In the results section we presented evidence to support all hypotheses, thus establishing the validity of the ACHWM Summary Score and its Physical, Emotional and Mental Quadrant scores. The Spiritual Quadrant made a unique contribution to understanding the well-being in this population. Although there is no analogous PedsQL domain against which to compare Spiritual scores, we did find a moderate correlation with the rest of the ACHWM, which was predicted a priori, based on the importance of balance across the quadrants in the theoretical model that is the basis for the ACHWM. Thus, our preliminary findings support its validity.

This study has several limitations that are worthy of consideration. First, the reliability of this measure has yet to be documented. Since the PedsQL is known to be highly reliable and the ACHWM has a moderate correlation with the PedsQL, it is likely to have at least moderate reliability, and further testing is underway. Second, this study focused on one community, and therefore the relevance in other communities may need to be assessed locally. Despite these limitations, we are confident that the main conclusions of this study remain.

There are several other observations from this study that are important to discuss. This study is the first to report estimated means and standard deviations for the ACHWM. As such, it provides information important for the design of future studies using this measure. This paper also identified that the ACHWM was stable across the 8 to 18 year age range, but that there were differences between boys and girls. The mean score for girls was 6.0 points lower than for their male counterparts. This requires further exploration.

This study also identified high ceiling effects on the PedsQL (19 %) in comparison to the ACHWM and to previous studies. Previous studies of the PedsQL have primarily focused on clinical samples rather than the general population. Thus the ACHWM has the potential to be able to detect change in both directions due to changes both positive and negative in the community. Furthermore, the ACHWM is unique in including a series of questions reflecting spirituality, which is a critically important domain in Aboriginal health frameworks. Moreover, this study is one of the first to include a specific mechanism for supporting children whose responses suggest a need for further assessment. This is an important innovation. These findings suggest that the ACHWM is well suited to its objective of gathering local population data to inform community health planning.

During the course of this study we had a special celebration of the project with the children, youth, and community members in Wikwemikong. During the celebration, the children gave this measure an Ojibway name, *Aaniish Naa Gegii*, meaning “how are you?”.

This measure is now ready for implementation, and will contribute to a better understanding of the health of Aboriginal children.

## Abbreviations

ACHWM: Aboriginal Children’s Health and Well-being Measure

## Footnotes

1 Note: We have elected to use the term *domains* to refer to scales within the PedsQL to avoid confusion between *summary scores* and *scale scores*.
